# Bioactive and Elastic Emulsion Electrospun DegraPol Tubes Delivering IGF-1 for Tendon Rupture Repair

**DOI:** 10.3390/ijms241210272

**Published:** 2023-06-17

**Authors:** Julia Rieber, Gabriella Meier-Bürgisser, Iris Miescher, Franz E. Weber, Petra Wolint, Yang Yao, Esteban Ongini, Athanasios Milionis, Jess G. Snedeker, Maurizio Calcagni, Johanna Buschmann

**Affiliations:** 1 Division of Plastic Surgery and Hand Surgery, University Hospital Zurich, Sternwartstrasse 14, 8091 Zurich, Switzerland; julia.rieber@usz.ch (J.R.); gabriella.meierbuergisser@usz.ch (G.M.-B.); iris.miescher@usz.ch (I.M.); petra.wolint@usz.ch (P.W.); maurizio.calcagni@usz.ch (M.C.); 2 Oral Biotechnology & Bioengineering, Center for Dental Medicine, Cranio-Maxillofacial and Oral Surgery, University of Zurich, 8032 Zurich, Switzerland; franz.weber@zzm.uzh.ch; 3 Department of Health Sciences & Technology & Department of Materials, Schmelzbergstrasse 9, LFO, 8092 Zurich, Switzerland; yang.yao@hest.ethz.ch; 4 Orthopaedic Biomechanics, University Clinic Balgrist, Forchstrasse 340, 8008 Zurich, Switzerland; esteban.ongini@hest.ethz.ch (E.O.); jess.snedeker@hest.ethz.ch (J.G.S.); 5 Laboratory of Thermodynamics in Emerging Technologies, Department of Mechanical and Process Engineering, ETH Zurich, 8092 Zurich, Switzerland; athanasios.milionis@ltnt.iet.mavt.ethz.ch

**Keywords:** Differential Scanning Calorimetry (DSC), emulsion electrospinning, Fourier Transform Infrared Spectroscopy (FTIR), gene expression, insulin-like growth factor-1 (IGF-1), release kinetics, scanning electron microscopy (SEM)

## Abstract

Tendon injuries can result in two major drawbacks. Adhesions to the surrounding tissue may limit the range of motion, while fibrovascular scar formation can lead to poor biomechanical outcomes. Prosthetic devices may help to mitigate those problems. Emulsion electrospinning was used to develop a novel three-layer tube based on the polymer DegraPol (DP), with incorporated insulin-like growth factor-1 (IGF-1) in the middle layer. Scanning electron microscopy was utilized to assess the fiber diameter in IGF-1 containing pure DP meshes. Further characterization was performed with Fourier Transformed Infrared Spectroscopy, Differential Scanning Calorimetry, and water contact angle, as well as through the assessment of mechanical properties and release kinetics from ELISA, and the bioactivity of IGF-1 by qPCR of *collagen I*, *ki67,* and *tenomodulin* in rabbit Achilles tenocytes. The IGF-1-containing tubes exhibited a sustained release of the growth factor up to 4 days and showed bioactivity by significantly upregulated *ki67* and *tenomodulin* gene expression. Moreover, they proved to be mechanically superior to pure DP tubes (significantly higher fracture strain, failure stress, and elastic modulus). The novel three-layer tubes intended to be applied over conventionally sutured tendons after a rupture may help accelerate the healing process. The release of IGF-1 stimulates proliferation and matrix synthesis of cells at the repair site. In addition, adhesion formation to surrounding tissue can be reduced due to the physical barrier.

## 1. Introduction

There have been many improvements in tendon rupture repair during the last decades [1,2,3]. The most common surgical procedures used for Achilles tendon repair are either minimally invasive or include percutaneous techniques [4]. Nevertheless, optimization approaches are still welcome because the usual, slow healing process, over 3 to 6 months, often does not lead to full functional recovery and strength [5]. The biomechanical inferiority of tendons undergoing healing requires support by a prosthesis [6] or other external cues that help to accelerate tissue regeneration [7].

Among different strategies, growth factors have been extensively studied to support tendon healing. For example, platelet-derived growth factor-BB (PDGF-BB) is a promising candidate [8] and has been reported to improve the biomechanical outcome of a fully transected and sutured rabbit Achilles tendon three weeks post-surgery [9]. Transforming growth factor-β (TGF-β), as a further important growth factor during tendon healing [10], has been reported to be of favorable influence, particularly when applied at the interface to the bone [11]. In addition, Insulin-like growth factor 1 (IGF-1) has recently gained attention with respect to Achilles tendon defect healing in a rodent model, where clay nanoparticles were used with absorbed IGF-1 and assembled into micro particles, which were physically trapped within hydrogel networks, providing a slow and steady release of this growth factor [12].

The growth factor IGF-1 is a protein produced through stimulation by the growth hormone (GH) in the liver [13,14]. It is known to regulate both anabolic and catabolic pathways in skeletal muscle [15], aid ageing processes (together with GH), and act synergistically with PDGF-BB in cell cultures aimed at tendon tissue engineering [16]. Moreover, several approaches including IGF-1 in tendon healing have been successful [17], i.e., reaching earlier functional recovery of a rat’s Achilles tendon [18] or a rat’s rotator cuff tendon [19], resulting in higher cell proliferation and collagen content in IGF-1-treated equine flexor tendinitis [20] or increased tendon biomechanics in rabbit patellar tendons in combination with TGF-β [21]. In addition, activation of the IGF1R (receptor for IGF-1) and MAPK signaling by a phytoestrogen has recently been reported to support tendon healing in a rat Achilles tendon model [22], demonstrating the positive effect of these signaling cascades that are also activated through IGF-1 binding to IGF1R. 

Considering these aspects, we developed an electro-spun elastic three-layer polymer tube with a sustained IGF-1 release, envisioned to be applied over a sutured tendon rupture. The fabrication of this device has been based on our previous studies, where we have developed a bioactive two-layer tube with a sustained release of PDGF-BB [9,23]. Compared to the 24.3 kDa-protein PDGF-BB, IGF-1 with 7.6 kDa [13,14] is rather small, so that release kinetics had to be optimized first by designing a multi-layer tube where the middle of three layers contains the bioactive component, while the two outer layers only consist of the polymer DegraPol (DP).

Here, we present the development and fabrication, as well as the in-depth characterization, of such an IGF-1 containing prosthesis in the form of a three-layer emulsion electro-spun DP tube, providing SEM, FTIR, DSC, water contact angle, and biomechanics, respectively. The objective of this study was to optimize IGF-1 release kinetics as a function of protein carrier serum albumin concentration and to verify the bioactivity of the released factor IGF-1 by qPCR when supplemented to rabbit Achilles tenocyte culture. As it has been reported that IGF-1 can be stored for more than 7 months in 20% serum at −80 °C [24], we determined the impact of long-term storage conditions (10 months) at −20 °C and at room temperature on the release kinetics of the novel implant, intending to pave the way of such a medical off-the-shelf device into the potential future clinical setting.

## 2. Results

### 2.1. Scanning Electron Microscopy and Fiber Thickness

**Figure 1:**
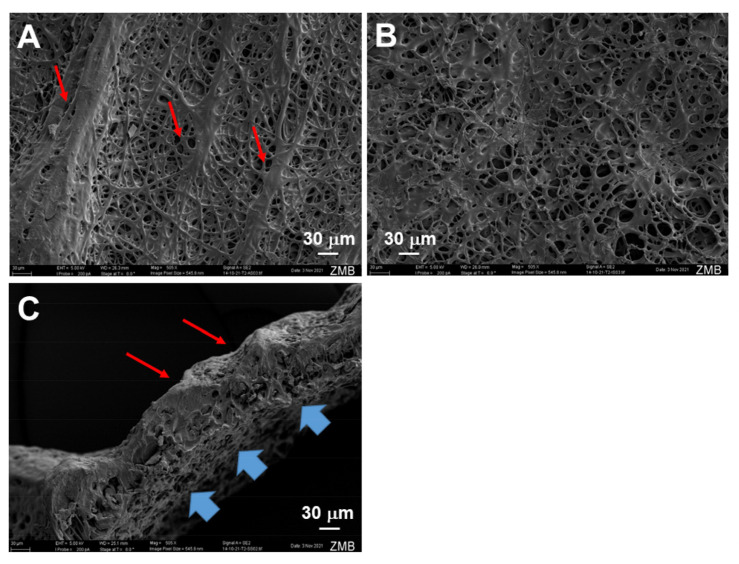
Scanning electron microscopy images of pure and IGF-1 containing tubes. Outer surface of emulsion electro-spun tube, exhibiting grooves (red arrows, (**A**)); inner surface of emulsion electro-spun tube with rather smooth surface (no grooves, (**B**)); cross-section of emulsion electro-spun tube showing grooves on outer surface (red arrows) and the smooth, but still porous, structure on the inner surface that faced the flat metal target during previous electrospinning (blue arrows, (**C**)). For the intended use in vivo, such tubes will be flipped so that the groove-like structure faces the repaired tendon, which makes the tube stay better in place due to the higher sliding friction coefficient of the grooved side [25].

Typical electro-spun tubes displayed different surface structures when the inner and outer surfaces were compared (Figure 1). The inner surface facing the metal rod as a target was smooth, while the outer surface exhibited a groove-like structure.

**Figure 2:**
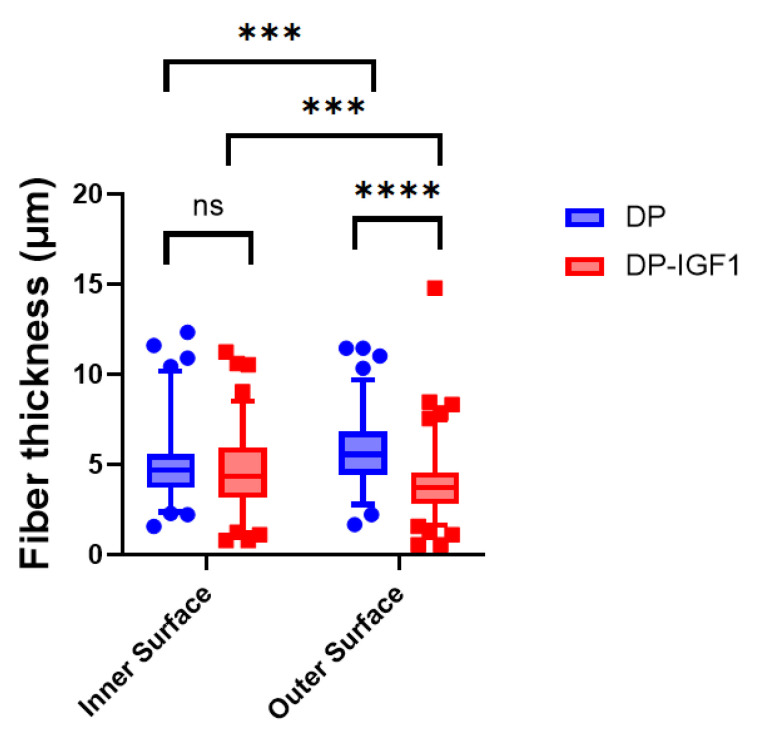
Fiber thickness of electro-spun meshes for pure DP meshes and emulsion electro-spun tubes containing IGF-1(DP-IGF1). For all tubes containing IGF-1, either in the middle layer or on the outer surface, the inner surface always consists of pure DP. Number of tubes n = 3. A nonparametric Kruskal–Wallis test was performed for each surface to determine significance in median difference. Data are shown as box and whisker plots with interquartile range and 95% confidence interval. *p*-values ≤ 0.05 were considered significant and denoted with *p* ≤ 0.001 (***); for *p* ≤ 0.0001 (****), and for non-significant (ns). Number of fibers used to assess diameters; inner surface DP n = 48 and DP-IGF-1 n = 141; outer surface DP n = 65 and DP-IGF-1 n = 167, respectively.

The fiber thickness of pure and emulsion electro-spun tubes, containing IGF-1 in a water-in-oil emulsion, did not differ much and was, on average, approximately 5 µm (Figure 2). While on the inner surface of both DP and IGF-1 tubes, the fiber thickness was not significantly different. On the outer surface (with the grooves), emulsion electro-spun tubes had a significantly lower fiber thickness compared with pure DP tubes.

### 2.2. Mechanical Properties

**Figure 3:**
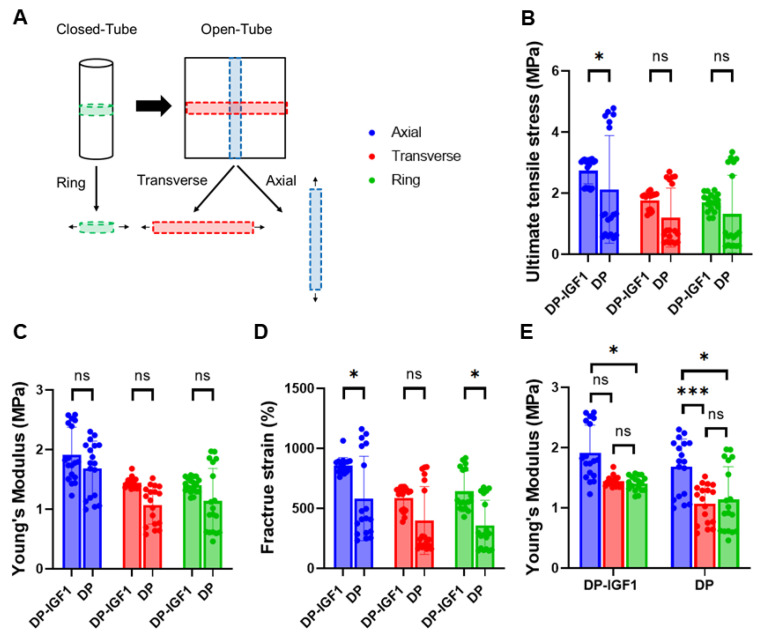
Mechanical properties of electro-spun tubes. Overview of how pieces were cut to assess mechanical properties in transverse and axial directions (**A**); ultimate tensile stress (**B**); Young’s modulus (**C**); fracture strain (**D**); Young’s modulus for IGF-1 (one layer) and pure DP tubes separately (**E**). The data are shown as mean and SD with individual values. n = 6. Mechanical properties were each compared by Kruskal–Wallis test, *p*-values ≤ 0.05 were considered significant and denoted with (*); for *p* ≤ 0.001 (***);and for non-significant (ns).

The mechanical properties of pure DP tubes and IGF-1 tubes were evaluated in both the transverse and axial directions due to the inherent anisotropy (grooves) of the material. The biomechanics in the transverse direction were assessed in two different ways: once as a ring piece (closed tube), and once as a band (opened tube). (Figure 3A). Ultimate tensile stress and Young’s modulus of elasticity were significantly higher for IGF-1-containing tubes compared with pure DP tubes (Figure 3B,C). The IGF-1 emulsion electro-spun tubes exhibited significantly higher fracture strains in the axial direction (Figure 3D). To evaluate the anisotropy of the material, differences in the ultimate tensile stress between axial and transverse directions were compared for each kind of tube separately (Figure 3E).

### 2.3. Fourier Transformed Infrared Spectroscopy and Differential Scanning Calorimetry

**Figure 4:**
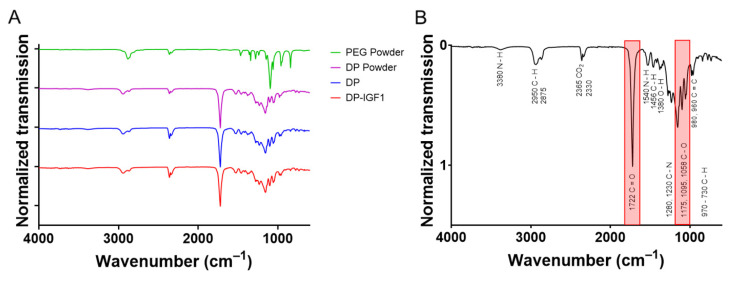
The FTIR assessment of IGF-1 containing tubes and pure DP tubes (n = 4) distributed on the y-axis as matter of presentation. As for electrospinning of DP and DP-IGF-1 tubes, a PEG layer is first produced, which is dissolved upon removal from the metal rod; the spectrum of PEG is added for comparison (**A**). For FTIR spectra of each single tube, see Figure S2. The FTIR assessment of DP tube with corresponding wavenumbers and chemical bonds, with most prominent peaks highlighted in red (**B**).

The FTIR spectra were taken of four DP tubes with incorporated IGF-1, four pure DP tubes, and DP powder, respectively (Figure 4). For comparison, the spectrum of polyethylene glycol (PEG) was assessed. The corresponding spectra of DP powder, pure DP tube, and IGF-1-containing tubes looked very similar; with prominent peaks at 1720 cm^−1^ for the C=O double bond and 1150 cm^−1^ for the C–O single bond, leading to similar ratios of C=O to C–O intensities (Figure S1). In contrast, the C–O single bonds in PEG had a lower wavenumber than in the DP, with 1070 cm^−1^. As DP exhibits many bands in the range of 900–1250 cm^−1^, it cannot be concluded that the C–O bonds originating from leftover PEG is visible in the DP samples due to overlay with other bands.

**Figure 5:**
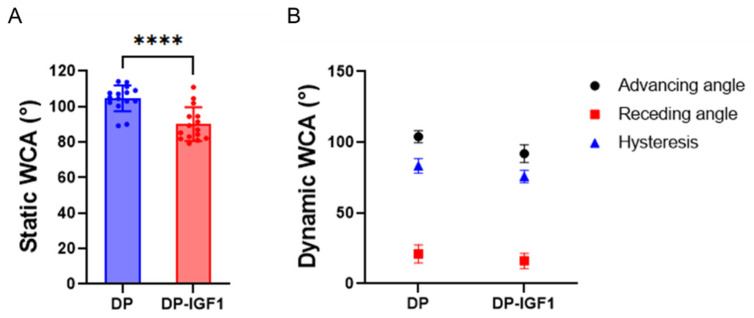
Static water contact angle (static WCA) of the inner surface for pure DP fiber mesh and emulsion electrospun DP fibers containing IGF-1 (**A**) and dynamic WCA, hysteresis = advancing WCA—receding WCA (**B**). WCAs for emulsion electro-spun tubes were determined to be statistically significantly lower than for pure DP tubes. For static WCA and dynamic WCA, five and three measurements, respectively, were averaged per sample, n = 3. The data are shown as mean and SD. The static contact angles were compared with an unpaired *t*-test; with *p* ≤ 0.0001 (****).

**Figure 6:**
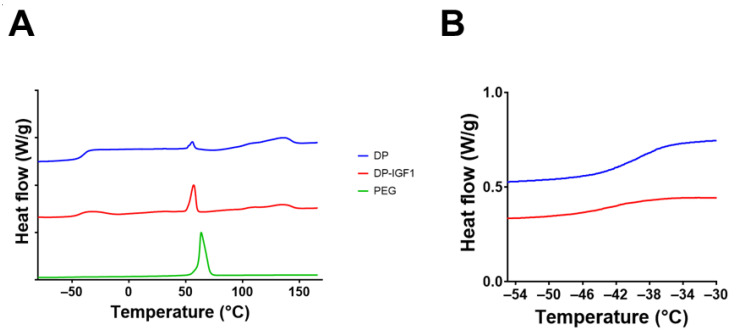
DSC assessment of IGF-1-containing tubes and pure DP tubes (**A**) with detail of glass transition region (**B**). For comparison, DSC of PEG is shown. Note that individual lines are distributed on the y-axis with an interval of 1 as a matter of presentation in A, whereas in B, the direct comparison is shown.

Furthermore, the assessment of static water contact angles (WCA) revealed significant lower WCA for electrospun IGF-1-containing fiber meshes compared with pure DP meshes (Figure 5, indicating that emulsion electrospun meshes are more hydrophilic than pure DP meshes). Moreover, DSC spectra were taken from DP tubes, DP-IGF-1 tubes, and PEG, respectively. As can be observed, DP- and IGF-1-containing tubes exhibit a melting point at 130 °C (Figure 6A; for each single tube, Figure S3) and a glass transition point at approximately −42 °C (Figure 6B), respectively. The prominent melting point of PEG at 60 °C is reflected in DP-IGF-1, and in pure DP tubes if washing is incomplete. In contrast to FTIR (Figure 4), DSC spectra of DP and DP-IGF-1 tubes can be used to check for complete PEG removal. 

### 2.4. Release Kinetics

**Figure 7:**
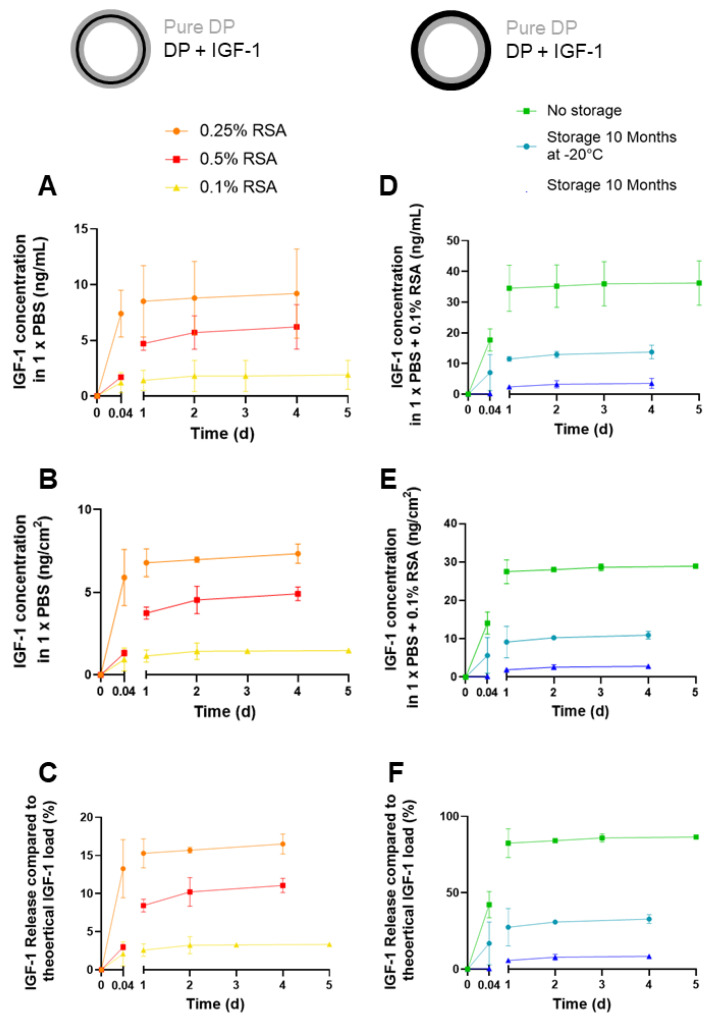
Cumulative release kinetics of IGF-1 as a function of rat serum albumin (RSA) in 3-layer tubes (**A**–**C**) and different storage conditions assessed for 2-layer tubes (**D**–**F**); in ng/mL (**A**,**D**), in ng/cm^2^ of scaffold (**B**,**E**) and in% of total load (**C**,**F**). Note that IGF-1 is released in lower amounts from 3-layer tubes, although in a more controlled way, while IGF-1 shows a burst release in 2-layer tubes. However, to compare storage conditions, it clearly demonstrates that *no storage*, i.e., freshly produced samples, is best with regards to the amount of growth factor released, while storage for 10 months at room temperature exhibited the lowest amounts of released IGF-1. Schematic overview of each tube is given; pure DP layer depicted in grey, IGF-1 layer in black. 0.004 d equals 1 h. Data are shown as mean and SD. For cumulative release curve of IGF-1 up to 42 days, please see Figure S5.

Release kinetics of freshly produced three-layer tubes were assessed for different rabbit serum albumin (RSA) concentrations. RSA is used as a carrier protein. Between two pure DP layers, one IGF-1 emulsion electro-spun layer was produced with 0.1, 0.25, and 0.5% RSA, respectively. Figure 7A demonstrates that the highest amounts of IGF-1 were released with 0.25% RSA, while the retention of IGF-1 within the middle layer was most prominent at 0.1% RSA, and the obtained release for 0.5% was in between. Compared to 0.1% RSA, the density of electro-spun meshes was lower at 0.5% RSA (Figure S4).

In addition, release kinetics of two-layer tubes (one layer with IGF-1 (0.1% RSA), one layer with pure DP fibers) were assessed immediately after production (no storage), and after 10 months of storage at either room temperature (RT) or −20 °C, respectively (Figure 7B). As can be seen, no storage leads to the highest amounts of released IGF-1, followed by storage at −20 °C and with comparably low amounts of released GF when tubes were stored at RT.

### 2.5. Bioactivity of IGF-1: Gene Expression of Collagen I, ki67, and Tenomodulin

**Figure 8:**
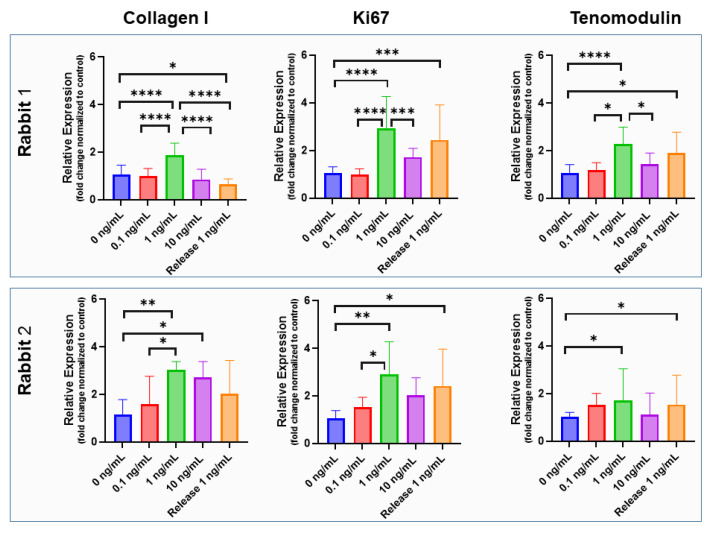
Bioactivity of incorporated IGF-1. Gene expression of rabbit Achilles tenocytes exposed to supplemented IGF-1 in concentrations of 0, 0.1, 1 and 10 ng/mL as well as exposed to IGF-1 released from emulsion electro-spun meshes (concentration 1 ng/mL; denoted as *Release 1 ng/mL*) for two rabbits (upper and lower row) and three markers (*collagen I*, *ki67* and *tenomodulin*); assessed for a 3-day cell culture. The data are shown as mean and SD, *p*-values ≤ 0.05 were considered significant and denoted with (*); for *p* ≤ 0.01 (**); for *p* ≤ 0.001 (***); for *p* ≤ 0.0001 (****). For impact of IGF-1 supplementation to rabbit Achilles tenocyte culture on cell morphology, see Figure S6.

The gene expression of *collagen I* in rabbit tenocytes of two different rabbits showed an increase from 0 to 1 ng/mL and a decrease from 1 ng/mL to 10 ng/mL of supplemented IGF-1 (Figure 8). Differences between 1 ng/mL supplemented IGF-1 and 0 ng/mL (control) were significant in tenocytes harvested from both rabbits, but the *collagen I* expression of formerly incorporated and released IGF-1 in a concentration of 1 ng/mL was not significantly different from the control, although a trend towards increased *collagen I* expression was found for Rabbit No. 2. For *ki67* and *tenomodulin*, bioactivity of released IGF-1 was confirmed by significant increases compared to the control in both rabbits, shown by the significant upregulation of both markers when stimulated by supplemented IGF-1. Both 1 ng/mL supplemented, as well as 1 ng/mL released IGF, induced approximately the same effect, verifying the maintained bioactivity of the protein after the electrospinning process.

## 3. Discussion

Tendon injuries and ruptures often result in two major problems during the healing process [26,27]. On the one hand, due to immobilization during early rehabilitation, adhesion formation to the surrounding tissue may limit the range of motion [28], leading to work disabilities and impeding daily activities. On the other hand, the biomechanically inferior repair tissue is prone to re-rupture [29], particularly in cases where high loads are applied too early after injury, caused by incautious or abrupt motions.

Hence, prosthetic devices that can be easily applied during reconstructive surgery are investigated. In this regard, one viable option is a bioactive, biodegradable, elastic, and surgeon-friendly small tube that is employed over the conventionally sutured tendon like a sleeve, as we have previously reported for the polymer tube DP with incorporated PDGF-BB [9,23]. Such tubes have been shown not only to decrease the adhesion formation by roughly 20% [30], but also significantly increase biomechanics of extracted tendons three weeks post-surgery [23]. As a result, re-rupture may be avoided at least to some extent. In an attempt to further optimize such implant materials, we focused on the incorporation of the growth factor IGF-1 in this study.

The growth factor IGF-1 is a small protein reported to support tenocyte proliferation in vitro [31], DNA synthesis, and protein synthesis, particularly Collagen I [32]. In addition to these proliferative and matrix stimulating effects when applied as a single factor, IGF-1 has been reported to act synergistically when applied in combination with PDGF-BB [33]. It has been reported that a combination of 100 ng/mL IGF-1 with 50 ng/mL PDGF-BB led to the highest proliferation level in a dose–response curve of tenocyte in-vitro cultures [33]. Moreover, 100 ng/mL of IGF-1, combined with 10 ng/mL of bFGF and 100 ng/mL of PDGF-BB, have been reported to act synergistically on adipose-derived stem cells intended to repopulate a hydrogel envisioned for tendon repair [34].

As the growth factor IGF-1 (7.6 kDa) is much smaller than PDGF-BB (24 kDa), our previous fabrication protocols of emulsion electrospinning [23] had to be adapted. Initially, we have fabricated an IGF-1-containing DP tube with two layers, analogously to the one reported earlier for PDGF-BB [23]. However, release kinetics were not optimum because IGF-1 was released instantly within the first hour (burst release [35]). Therefore, we incorporated IGF-1 in the middle layer of a novel three-layer tube, with the outer two layers consisting of pure DP resembling a sandwich, while the IGF-1 layer was fabricated by emulsion electrospinning of a water-in-oil emulsion with aqueous IGF-1 in DP (dissolved in organic solvents). For the release, the distance to be covered by IGF-1 was longer. Upon this optimizing step, release kinetics turned out to have a more prolonged, sustained pattern over several days (Figure 7).

The SEM images of such electro-spun meshes exhibited a smooth surface facing the target, and a grooved surface, on the outside (Figure 1), leading to the observed anisotropic mechanics (Figure 3). However, SEM imaging did not show large differences with respect to fiber diameters when the inside surface was compared to the outside surface (Figure 2), with approximately a diameter of 5 μm for pure- and IGF-1-containing fibers, respectively. Similar fiber thickness has been reported for other electro-spun meshes, such as cyto-compatible and biodegradable fibrous wound dressings [36]. The large standard deviation results from the high variability of fiber thickness. However, there was a significantly lower fiber thickness for IGF-1 emulsion electro-spun tubes on the outer surface when compared to pure DP tubes. As the voltage and flow rate were kept constant for the fabrication of pure DP and IGF-1 tubes [23], the smaller fiber diameters assessed for emulsion electro-spun fibers have to be attributed to the changes in the surface tension and viscosity of the polymer solutions. Compared with pure DP solutions, the polymer jet of a water-in-oil emulsion is less stable and subject to earlier splitting, resulting in smaller fiber thickness on the target, as it was observed here. These findings stand in accordance with other emulsion electrospinning studies, where, for example, adding bovine serum albumin into the aqueous phase increased conductivity and elongated the polymer jet to result in thinner fibers compared with the pure polymer fibers (no aqueous phase) [37]. On average, thinner fibers were also obtained via increasing the percentage of water in a water-in-oil electrospinning study [38].

The mechanical analysis confirmed the anisotropic behavior of the electro-spun tubes, with higher strain at break, ultimate stress, and Young’s modulus for axial direction compared with transverse direction [23]. Interestingly, IGF-1-containing tubes exhibited significantly different mechanical properties compared with pure DP tubes. The fracture strain was significantly higher in emulsion electro-spun tubes, implying that the emulsion electro-spun fibers lead to lower macromolecular friction when stretched, which is caused by the tiny water droplets within such fibers (Figure 3D).

The ultimate tensile stress was increased by roughly a factor of 1.3 in IGF-1 tubes compared to pure DP tubes (Figure 3B), which may be explained by potential crystallization processes induced by the presence of water droplets within fibers composed of the block-co-polymer DP. It is speculated that the hard segment composed of large units (poly-hydroxy-butyrate) may be impacted by the water droplets so that hydrolysis locally leads to smaller polymer units with different (potentially harder) characteristics. Finally, the elastic modulus was found to be increased in IGF-1-containing tubes when compared to the pure DP tubes (Figure 3C), corresponding with the findings of significantly higher ultimate stress discussed above.

In addition to these differences between the two kinds of tubes, there were also significantly different mechanics for transverse and axial directions, respectively. The properties in the axial direction, where the grooves were parallel to, turned out to be significantly higher (Figure 3E). The grooves on the outside led to an amplification of the forces needed to break the material, as they consist of practically pore-less entities of thick and condensed electro-spun fibers. Hence, we were not surprised to find a prominent anisotropy.

The tubes were further characterized by FTIR (Figure 4 and Figure S2). As expected, no obvious differences were found between pure DP tubes and IGF-1-containing emulsion electro-spun tubes. The characteristic band for C=O double bonds at 1720 cm^−1^ was verified for both pure and emulsion electro-spun tubes. This bond is typical for polyesters as well as polyurethanes, ranging from 1730–1690 cm^−1^ [39]. DP is a block-co-polymer, with basic units of polyester urethanes, originally developed for bone tissue engineering [40]. Moreover, DP and IGF-1 tubes displayed the same multiple band pattern in the range of 1250–900 cm^−1^, with a prominent band at 1150 cm^−1^, which is characteristic for the C-O single bonds. Repetitive fabrication of such electro-spun tubes led to the same FTIR spectra, as shown in three tubes (Figure S2). When these spectra were compared to pure PEG, they were used as a first layer and then dissolved in order to easily detach the tube from the metal rod. However, there was a clear absence of the band at 1720 cm^−1^. In addition, there was a difference in the multiple bands in the range of 1250–900 cm^−1^, caused by the absence of the C=O bond next to the C–O single bonds (polyether instead of polyester), which may lead to a shift of the C–O wavenumber to lower values compared to esters. Aliphatic ethers exhibit an asymmetric stretching in the range of 1150–1085 cm^−1^, while aliphatic esters have wavenumbers of 1210–1163 cm^–1^ for two coupled asymmetric stretching [41].

As expected, the static WCA was lower for the water-in-oil emulsion electro-spun scaffold, including IGF-1, compared with the pure DP fiber mesh (Figure 5). This implies a significantly higher hydrophilicity for the emulsion electro-spun mesh, which is attributed to the small water droplets incorporated within the polymer fibers. Accordingly, the dynamic WCAs (both advancing and receding) were lower for the IGF-1 samples compared to the pure DP scaffolds, although not significantly different. A large contact angle hysteresis was found for both materials (70–80°), implying a quite heterogeneous surface of both electro-spun materials [42], which was confirmed by SEM (Figure 1).

Furthermore, thermograms were assessed for the different materials (Figure 6 and Figure S3). The DSC spectra for DP tubes and IGF-1-containing emulsion electro-spun DP tubes looked very similar, with a melting point of approximately 130 °C, which confirms previous findings for DP powder (129 °C) and electro-spun pure DP mesh (134 °C) [9]. In contrast, pure PEG has a melting point of 70 °C. However, when PEG impurities are in electro-spun DP meshes, the melting shifts to slightly lower melting points, i.e., 65 °C [9], which was confirmed here (Figure 6A). As for the glass transition point, it was found at approximately −38 °C for DP [43].

In-vitro release kinetics of IGF-1 released from scaffolds were assessed for different formulations (Figure 7A); similar experiments had been performed earlier with model biomolecules that are not bioactive and had low molecular weight (fluorescein, 376.27 g/mol) and high molecular weight (FITC-BSA = FITC labelled bovine serum albumin, 66 kDa), respectively, which can be regarded as not including bioactive control compounds [23]. Rabbit serum albumin (RSA) was used as a carrier protein for IGF-1 because, otherwise, it would be degraded [44,45]. Rabbit serum albumin but not BSA was utilized in view of planned rabbit Achilles tendon in-vivo experiments in the future. Different concentrations of RSA led to different amounts of IGF-1 released from the middle layer of three-layer tubes, with a concentration of 0.25% RSA resulting in the highest amount. Lower concentrations (0.1% RSA) led to a release of only roughly 20% of what was released at 0.25% RSA, indicating suboptimum stabilization of IGF-1. On the other hand, a 0.50% RSA concentration in the aqueous phase of the emulsion resulted in a stabilization and retention within the fibers that was too strong to ideally release the growth factor, which was retained inside the fibers. At any RSA concentration, most of the IGF-1 was released within one day, followed by a sustained release until Day 4. However, in-vivo application will have different release kinetics, as the complex situation with enzymes in the wound bed accelerate DP degradation and, therefore, release more IGF-1 in a different, time-dependent way. This has been exemplified by an earlier lipase degradation of DP scaffolds with incorporated PDGF-BB [9].

Furthermore, as the bioactive tubes are envisioned to be used in clinics, the impact of storage temperature has been assessed (Figure 7B). The release kinetics of IGF-1 incorporated in one layer of two-layer tubes indicated that fresh conditions, in other words, just after electrospinning, resulted in the highest amounts of released growth factor. Nevertheless, if storage is considered, as illustrated for a period of 10 months, it is better to store the scaffolds at −20 °C than at room temperature (RT). The impact of storage conditions has been assessed and reported in another study where ascorbic acid was incorporated in DP fiber meshes. However, these samples had been stored for only 1 week. Nevertheless, a similar trend was found with the highest amounts of ascorbic acid released from fresh samples and decreasing amounts with storage at descending temperatures in the order of RT, 4 °C, and −20 °C, respectively [46].

Gene expression of *collagen I* has been reported to be increased in equine tenocyte cultures, with 2- to 3-fold increases under 10 and 100 ng/mL IGF-1 [47]. Other reports have found a negligible impact of IGF-1 on *collagen I* expression in rat tail tenocyte culture for the same IGF-1 concentrations [48]. In our rabbit Achilles tenocyte 3-day culture, supplemented with IGF-1, we found a dose-dependent increase of *collagen I* gene expression at 0, 0.1 and 1 ng/mL, followed by a decrease at 10 ng/mL IGF-1 (Figure 8). Therefore, the bioactivity of released IGF-1 was tested for 1 ng/mL. However, the released IGF-1 did not induce a significant increase in *collagen I* gene expression after three days, although in one of the two rabbit donors, there was a trend towards higher *collagen I* expression compared to the control.

However, retained bioactivity of the growth factor IGF-1 could be demonstrated with gene expression of *ki67* and the typical tendon marker gene *tenomodulin*. For both markers, and for tenocytes isolated from rabbit Achilles tendons originating from two different rabbits, released IGF-1 protein had very similar impact than freshly added IGF-1 (the gene expression was significantly higher than in the control) (no IGF-1) (Figure 8). Although performing experiments at higher concentrations of IGF-1 than utilized here, Musson et al. have also reported increased *tenomodulin* expression in rat tenocyte culture after 3 days [48]. Thus, our findings go along with reports from literature and confirm the maintained bioactivity of IGF-1 after the electrospinning process.

Finally, cell culture experiments on Day 3 with different concentrations of IGF-1 revealed a significant change in morphology at 10 ng/mL IGF-1 (Figure S6). Very similar to the findings for PDGF-BB supplementation [30], cells exhibited smaller length–width ratios under IGF-1 supplementation. Such a reorganization of the cytoskeleton could be attributed to the activation of the PI3K/Akt-signaling pathway, as both PDGF-BB and IGF-1 cause phosphorylation of Akt through binding to their corresponding receptors, PDGFR and IGF1R, respectively [9,17].

Although the focus of this article lies on the fabrication and characterization of novel emulsion electro-spun implant materials with incorporated IGF-1 protein intended to support tendon rupture repair, there are still some limitations. First, cell–material interactions are missing here and could give insight into IGF-1 effects on cell adhesion, particularly with F-actin and vinculin stainings to characterize focal adhesion formation. In addition, changes in cell morphology resulted by IGF-1 release to seeded cells might be of interest and could be compared to results obtained in cell-culture plates (Figure S6). Second, an in-vivo assessment of these implant materials has to be performed in pre-clinical animal models in order to assess their suitability and their effects on tissue regeneration.

## 4. Materials and Methods

### 4.1. The Polymer DegraPol

For the synthesis of DP, the 25 wt% of poly (3-(R-hydroxybutyrate)-co-(ε-caprolactone)-diol (Mn = 2824 g mol^−1^) and 75 wt% of poly(ε-caprolactone)-diol-co-glycolide (15 mol% glycolide, 85 mol% ε-caprolactone) (Mn = 1000 g mol^−1^) were dissolved in 1,4-dioxane and dried until water content was below 20 ppm. The solution was cooled, and a stoichiometric amount of 2,2,4-trimethylhexane-diisocyanate (TMDI) was added. After 1 day, dibutyltin dilaurate (20 ppm) was added three times within 1 d in order to reach molecular weight of 100–110 kDa. The polymer was precipitated in cooled hexane isomers and purified with chloroform and silicagel 60 column (Fluka), followed by precipitation in cooled ethanol [23,25].

### 4.2. Incorporation of the Growth Factor IGF-1

The solutions were prepared 1 to 3 days before electrospinning. For each scaffold, a polyethylene glycol (PEG) (35 kDa, Sigma-Aldrich, Berlin, Germany #81310) solution was prepared by adding 1.5 g of PEG and 3.5 g of chloroform (Sigma–Aldrich, Germany, #132950). The DP solution was produced by adding 0.6 g of DP powder, 3.52 g of chloroform, and 0.88 g of 1,1,1,3,3,3-Hexafluoro-2-propanol (HFP, Sigma-Aldrich, Germany, #105228) into a glass with a screw cap. For the incorporation of recombinant human IGF-1 (PeproTech, Boston, MA, USA, #100-11-100UG), a total amount of 4 µg of IGF-1 dissolved in 400 µL of phosphate-buffered solution (PBS, BioConcept, Allschwil, Switzerland, #3-05F39-I) containing RSA (10 µg/mL IGF-1 and 0.25% RSA in PBS) was added drop-wise to the DP solution, while stirring for five minutes on the magnetic stirrer at 500 rpm. The solution was shortly mixed on the vortex and further emulsified in an ultrasonic bath for 15 min. The emulsion was filled in a 5-mL glass syringe (Huberlab, Aesch, Switzerland,# 3.7102.33) and used immediately for electrospinning. As this protocol for water-in-oil emulsion with IGF-1 was the same as used previously for PDGF-BB incorporation, homogenous distribution of water droplets containing IGF-1 within the DP fibers can be assumed [23].

### 4.3. Electrospinning

An in-house assembly was used for electrospinning, consisting of a DC high voltage supply (Glassman High Voltage Inc., High Bridge, NJ, USA), a needle holder, transporter, and syringe pump (SP210cZ, WPI, Berlin, Germany). The spinning head with a blunt end included a needle (1 mm inner diameter and 0.3 mm wall thickness) made of stainless steel (Angst and Pfister AG, Zürich, Switzerland). A metal rod with a length of 55 cm was mounted to the rotary motor (the Euro Star B rotary motor, IKA Labortechnik, Staufen im Breisgau, Germany) and served as collector. The electrospinning conditions for tube production were 1 mL/h as a flow rate, 1with 9.5-cm working distance between the spinning needle and the metal rod and 12.5-kV voltage applied. The collector rotated with 500 rpm. The room temperature was constant (22–23 °C), and the humidity varied between 25–35%. The needle that was transporting the DP solution was moving constantly sideways in both directions in a total range of 20 cm. A first layer of PEG was deposited on the metal rod to facilitate the detachment of the DP tube. Subsequently, the DP or DP with IGF-1 layers was electro-spun on top of the PEG layer. After detachment with 50% ethanol, the tubes were stored in a desiccator.

### 4.4. Release Kinetics of IGF-1 from Electrospun Mesh

To assess release kinetics of IGF-1, three samples of a 0.2-cm diameter tube with 0.5 cm of length were placed in separate low-binding micro tubes (Eppendorf, Switzerland, #022431064). Then, 500 µL of PBS with 0.1% RSA were added to each sample as release medium with subsequent incubation at 37 °C and shaking at 300 rpm. At each respective time point, the media was removed into a labeled Eppendorf, and the low-binding tubes were filled up with new medium. The release samples were stored at −20 °C until further usage. The IGF-1 release was quantified by the human Elisa Kit (abcam, Amsterdam, Netherlands, ab108873) following the manufacture’s protocol. Amount of IGF-1 was measured with an ELISA–Microplate–Reader (BioTeck, Cytation/5 imagine reader, Basel, Switzerland) at 450 nm wavelength, and 570 nm as wavelength correction. The values were represented as cumulative release over time (ng/mL). After incubation, the scaffolds were stored in the residual PBS solution at −20 °C.

### 4.5. Tenocyte Culture and Real-Time PCR

Rabbit tenocytes isolated from Achilles tendons of New Zealand white rabbits were used. The tenocytes were thawed and resuspended in culture medium (Ham’s F12 (biowest, Nuaillé, France #L0135-500) with 10% FBS (biowest, south America, Nuaillé, France, #S1830-500), 1% Penicillin/Streptomycin (Sigma, Israel, #P4333-100ML), and 1% glutamax (Life Technologies, Boston, MA, USA, #35050-038)). Tenocytes from Passages 2 and 3 (P2–P3) were used. The impact of supplementation of culture medium by recombinant human IGF-1 at concentrations of 0, 0.1, 1, and 10 ng/mL were tested, as well as released IGF-1 at a concentration of 1 ng/mL. 

In order to determine the effect of IGF-1 on the gene expression of tenocytes, rabbit tenocytes were seeded into six-well plates (Sigma, #SIAL0516, growth area per well: 9.6 cm^2^) with a density of 2 × 10^5^ cells/well in 2 mL of culture medium. Cells were allowed to attach overnight. Desired IGF-1 concentration was added to the medium at Day 0. Samples were cultured at 37 °C with 5% CO_2_ and collected after three days, where photos were also recorded to assess aspect ratios. Rabbit tenocytes from two donors were used, and qPCRs were performed in triplicates. At the respective time point, total RNA was isolated, using the RNeasy Plus Mini Kit (Qiagen, Hilden, Germany, #74104) with RNase-free DNase treatment (Qiagen, Hilden, Germany, #74104) following the manufacturer’s protocol. The purity and amount of RNA were measured with Nanodrop One (ThermoFisher, Boston, MA, USA, ND–ONE–W). For reverse transcription (RT), 500 ng of RNA were investigated in a reaction volume of 20 µL (SuperScript III Reverse Transcriptase, Thermo Fisher, # 18080085; Oligo(dT) 12-18 Primer, Thermo Fisher, # 18418012; RNase Inhibitor, Applied Biosystem; # N8080119, dNTP, Invitrogen, Waltham, MA, USA, #18427013) using a compact thermocycler (applied biosystems by Thermo Fisher, Singapore, #A37028) scientific. Real-Time PCR reactions were performed in technical triplicates with 4 µL of the resulting cDNA (cDNA was diluted 10-fold with water for the analyses in a reaction volume of 20 µL and 180 µL of water) using the Quant Studio 5 (Applied Biosystems, Boston, MA, USA, CYT5M) and Fast SYBR Green Master Mix (Thermo Fisher, Lithuania, #4385612). The samples were heated to 95 °C for 3 min, followed by 40 cycles of 95 °C for 3 s and 60 °C for 20 s. All rabbit primers, listed in Table S1, were synthesized by Microsynth, Balgach, Switzerland. Relative expression analysis was performed using the comparative 2^−∆∆CT^ method with 18S as a reference gene, which was stable over the conditions analyzed. Results are presented as fold change normalized to control, i.e., compared to the averaged samples cultivated without IGF-1 (normalized to 1) [49].

### 4.6. Scanning Electron Microscopy (SEM)

Samples were prepared to visualize the inner and outer surface and the cross section of the electro-spun mesh. The samples were fixed on the SEM-carrier with conductive double-sided adhesive. Coating and imaging were performed with equipment maintained by the Center for Microscopy and Image Analysis, University of Zurich. The samples were coated with a 10-nm film of platinum with a sputter coater (Safematic CCU-010). The examination was by SEM (Zeiss Gemini SEM 450, Zeiss, Jena, Germany) at a high voltage of 5 kV. Pictures were taken at a magnification of 507× and at a brightness of 49%. The detector was set to secondary electrons. The fiber diameter and the thickness of the tube wall were measured by ImageJ (1.53e/Java 1.8.0_172 (64-bit)) using the scale bar from the microscope image. For standardization of the wall thickness, the scheme of a clock was used, and SEM pictures were recorded at 2, 4, 6, 8, 10, and 12 o’clock, respectively. From each image, there were three random measurements. Per each SEM image, a diagonal line was drawn and all the fibers, crossed by the line, were measured.

### 4.7. Fourier Transformed Infrared Spectrometry (FTIR)

Fourier Transformed Infrared Spectroscopy was carried out on a Varian 640 Fourier Transform Infrared Spectrometer (FTIR) equipped with a Golden Gate-diamond ATR with temperature control. The spectra were collected in a wavenumber range of 600–4000 cm^−1^; the resolution was set to 4 cm^−1^. To obtain the spectrum, 64 scans were averaged. For comparison of the differently fabricated scaffolds, the ratio between the C=O peak at 1720 cm^−1^ and the C–O peak at 1175 cm^−1^ was calculated. For visual presentation of the data, normalization to the C=O peak at 1720 cm^−1^ was performed. DP powder was assessed, as well as pure DP tubes and different emulsion DP tubes, respectively. PEG was also measured to check for PEG contamination. Corresponding bonds were assessed by comparing the peaks to IR-spectrum table (Merck KGaA, Darmstadt, Germany).

### 4.8. Differential Scanning Calorimetry (DSC)

Thermal analysis of the samples was performed using a differential scanning calorimeter, DSC2500 (TA Instruments, New Castle, DE, USA). Samples were loaded into the machine in a weight range of 3 to 15 mg. The thermograms were measured from −90 °C to 170 °C, with a heating and cooling rate of 10 °C/min. Two heating cycles with an intermediate cooling cycle were collected. The glass transition point and the phase transition enthalpy were calculated with the software of the DSC (TA Instuments TRIOS v5.1.1.46527, New Castle, DE, USA). For comparison of different samples, only the second heating cycle was taken into consideration. DP powder was assessed, as well as pure DP tubes and different emulsion DP tubes, respectively. Pure PEG was included to check for PEG contamination in the DP samples.

### 4.9. Static and Dynamic Water Contact Angles

The water contact angle (WCA) for pure DP and DP emulsion electro-spun meshes with IGF-1 were measured. Static and dynamic contact angles on the surfaces were measured by a video-based optical contact angle measuring instrument OCA 35 Dataphysics, Germany. A drop volume of 5 µL was set according to reported WCA measurements [50]. The samples were placed under the syringe (1 mL) and filled with milli-Q water; a drop was then placed on the surface. The left and right angle formed between the water drop and the surface were measured. The mean of these angles was calculated and defined as WCA; five measurements were averaged per sample.

The advancing WCA was assessed in a similar way. An initial static drop (5 µL) was filled further with water; when the baseline (the area where the drop is in contact with the material) increased, the advancing angle was calculated. The receding angle was measured when the baseline decreased during removal of water. The contact angle hysteresis was calculated by subtracting the receding from the advancing angle [42]. For measuring the contact angle, a video of the process was recorded, and the left and right angles were measured by ImageJ (1.53e/Java 1.8.0_172 (64-bit)) and averaged. Three measurements were made from each sample (n = 3).

### 4.10. Mechanical Properties

For the measurement of the mechanical properties, pure DP and emulsion DP tubes, including IGF-1, were used. The stress/strain curves were measured using a uniaxial load test machine (Zwick Z010 with a 20N load cell). The axial and transverse directions were measured by using rectangular specimen cut from 6 mm tubes to 2 mm × 18 mm. Samples were clamped to a gauge length of 10 mm. For the measurements of tubes, they were cut in 2-mm pieces and clamped to a gage length of 8 mm. As the thickness of these samples was different, only material properties were assessed and compared. For all samples, a strain rate of 10 mm/min was applied until failure. The nominal strain [%], tensile force [N], specimen width [mm], and layer thickness [mm] were assessed. The strain at break [%], ultimate tensile stress [MPa], and Young’s modulus [MPa] were determined for every condition (n = 6).

### 4.11. Statistics

Data were analyzed by GraphPad Prism 9 (Version 9.4.0 GraphPad Software Inc., San Francisco, CA, USA). A Shapiro–Wilk test was used to test for normal distribution of the data. For comparing two groups, an unpaired *t*-test was used in case of normal distribution of data. Otherwise, a Wilcoxon test was applied. For analysis of more than two groups, the following tests were used. Where normally distributed data was confirmed, a one-way analysis of variance (ANOVA with Tukey’s multiple comparisons test) was used for pairwise comparison. If the data were not following normal distribution, a nonparametric Kruskal–Wallis test was used. *p*-values ≤ 0.05 were considered significant and denoted with (*); for *p* ≤ 0.01 (**); for *p* ≤ 0.001 (***); for *p* ≤ 0.0001 (****); and for non-significant (ns). Calculations of standard curves and other calculations were performed in Excel (2016 (16.0.5332.1000) MSO (16.0.5278.1000) 32-bit).

## 5. Conclusions

We have fabricated novel emulsion electro-spun meshes envisioned to be used in tendon rupture repair. They have the growth factor IGF-1 incorporated in the middle layer of a three-layer tube. Most of IGF-1 was released during the first day. However, a sustained release was followed-up to 4 days. The released IGF-1 was still bioactive, as demonstrated by significant upregulation of *ki67* and *tenomodulin*. The scaffolds were characterized in-depth, including fiber thickness (SEM), WCA, DSC, and FTIR, as well as mechanical properties of the anisotropic electro-spun meshes in the form of tube-like implants. They are then ready to be tested in vivo in a pre-clinical animal model to eventually reach the final aim of clinical application.

## Data Availability

Not applicable.

## Supplementary Materials

The following supporting information can be downloaded at: https://www.mdpi.com/article/10.3390/ijms241210272/s1.
