# Supplementary material for: Bioactive and Elastic Emulsion Electrospun DegraPol Tubes Delivering IGF-1 for Tendon Rupture Repair
Source: Int J Mol Sci. 2023 Jun 17;24(12):10272. doi: 10.3390/ijms241210272 (PMC10299220; doi:10.3390/ijms241210272)
Supplement: Supplementary file 1 [file ijms-24-10272-s001.zip › ijms-2419181-SI.pdf]

Supporting Information

Bioactive and Elastic Emulsion Electrospun DegraPol Tubes Delivering IGF-1 for Tendon Rupture Repair

Julia Rieber [1], Gabriella Meier-Bürgisser [1], Iris Miescher [1], Franz E. Weber [2], Petra Wolint [1], Yao Yang [3], Esteban Ongini [4], Athanasios Milionis [5], Jess G. Snedeker [4], Maurizio Calcagni [1] and Johanna Buschmann [1,*]

[1] Division of Plastic Surgery and Hand Surgery, University Hospital Zurich, Sternwartstrasse 14, 8091 Zurich, Switzerland; julia.rieber@usz.ch, gabriella.meierbuergisser@usz.ch, iris.miescher@usz.ch, petra.wolint@usz.ch, maurizio.calcagni@usz.ch

[2] Oral Biotechnology & Bioengineering, Center for Dental Medicine, Cranio-Maxillofacial and Oral Surgery, University of Zurich, Zurich 8032, Switzerland; franz.weber@zzm.uzh.ch

[3] Department of Health Sciences & Technology & Department of Materials, Schmelzbergstrasse 9, LFO, 8092 Zürich, Switzerland; yang.yao@hest.ethz.ch

[4] University Clinic Balgrist, Orthopaedic Biomechanics, Forchstrasse 340, 8008 Zurich, Switzerland; esteban.ongini@hest.ethz.ch; jess.snedeker@hest.ethz.ch

[5] Laboratory of Thermodynamics in Emerging Technologies, Department of Mechanical and Process Engineering, ETH Zürich, 8092 Zürich, Switzerland; athanasios.milionis@ltnt.iet.mavt.ethz.ch

* Correspondence: johanna.buschmann@usz.ch, +41 44 255 98 95

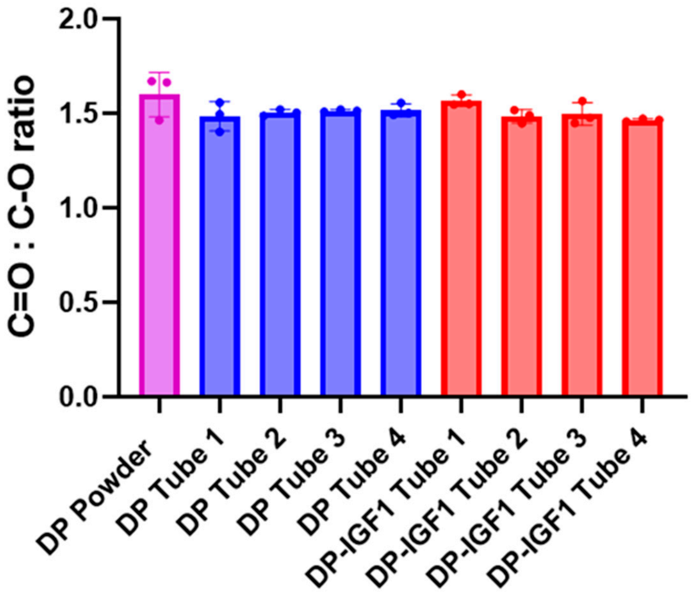

Figure S1: FTIR C=O to C-O band ratio assessed for DP powder, four pure DP tube and four DP tubes containing an emulsion electrospun layer with IGF-1. Data is shown as mean and SD with individual values, no significant difference in C=O : C-O ratio between individual tubes was detected with ANOVA.

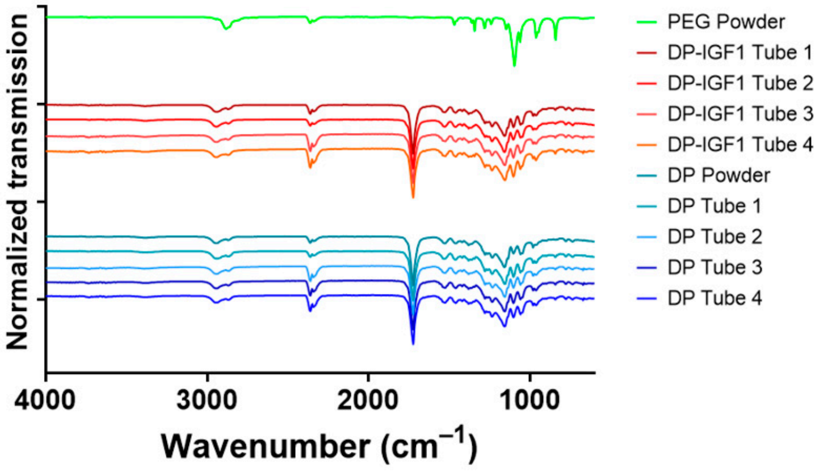

Figure S2: Further FTIR spectra, including the spectrum of pure PEG powder for comparison. For better comparability, the spectra are grouped in pure DP (bluish) and emulsion electrospun IGF-1 tubes (reddish).

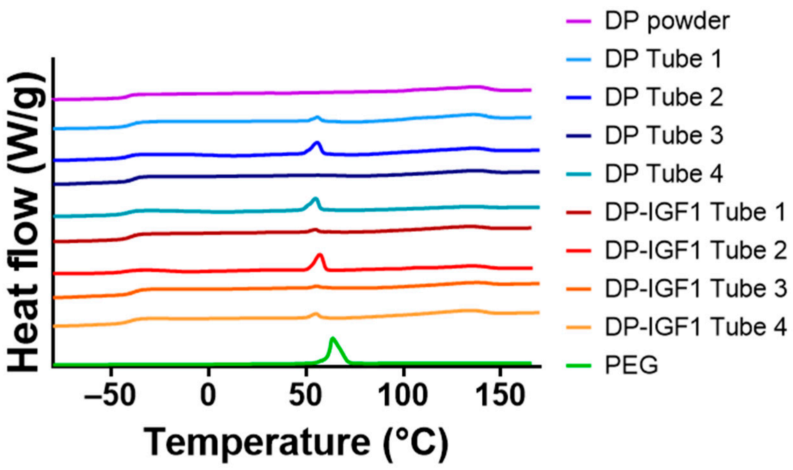

Figure S3: DSC measurements of DP and DP-IGF-1 tubes and pure PEG for comparison. For better comparability, the spectra are grouped in pure DP (bluish) and emulsion electrospun IGF-1 tubes (reddish).

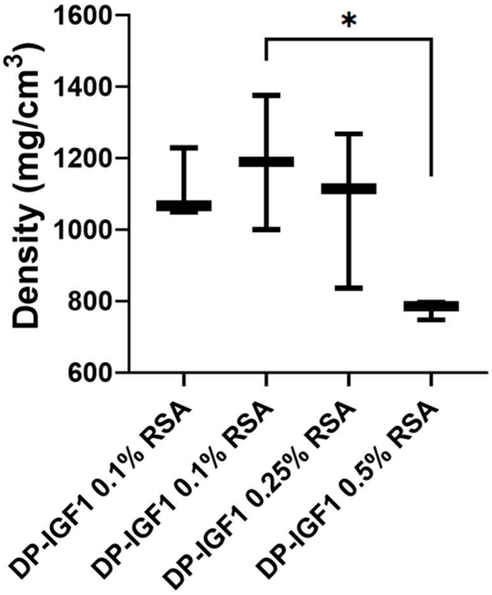

Figure S4: Density of electrospun meshes. Tubes 1 and 2 both had the same composition with 0.1 % RSA to stabilize IGF-1, while tube 3 included 0.25 % RSA and tube 4 0.5 % RSA, respectively. Data is shown as box and whisker plots with interquartile range and 95 % confidence interval. Significant difference between 0.1 % RSA tube 2 and 0.5 % RSA was found with one-way ANOVA. p-values < 0.05 were considered significant (*).

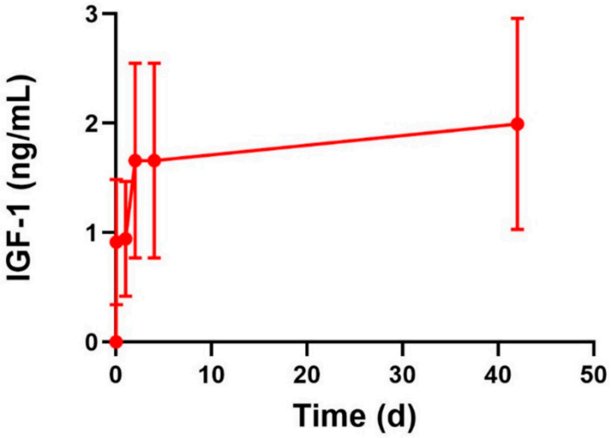

Figure S5: The IGF-1 release curve over 42 days. As can be seen, sustained release of IGF-1 up to 6 weeks can be achieved.

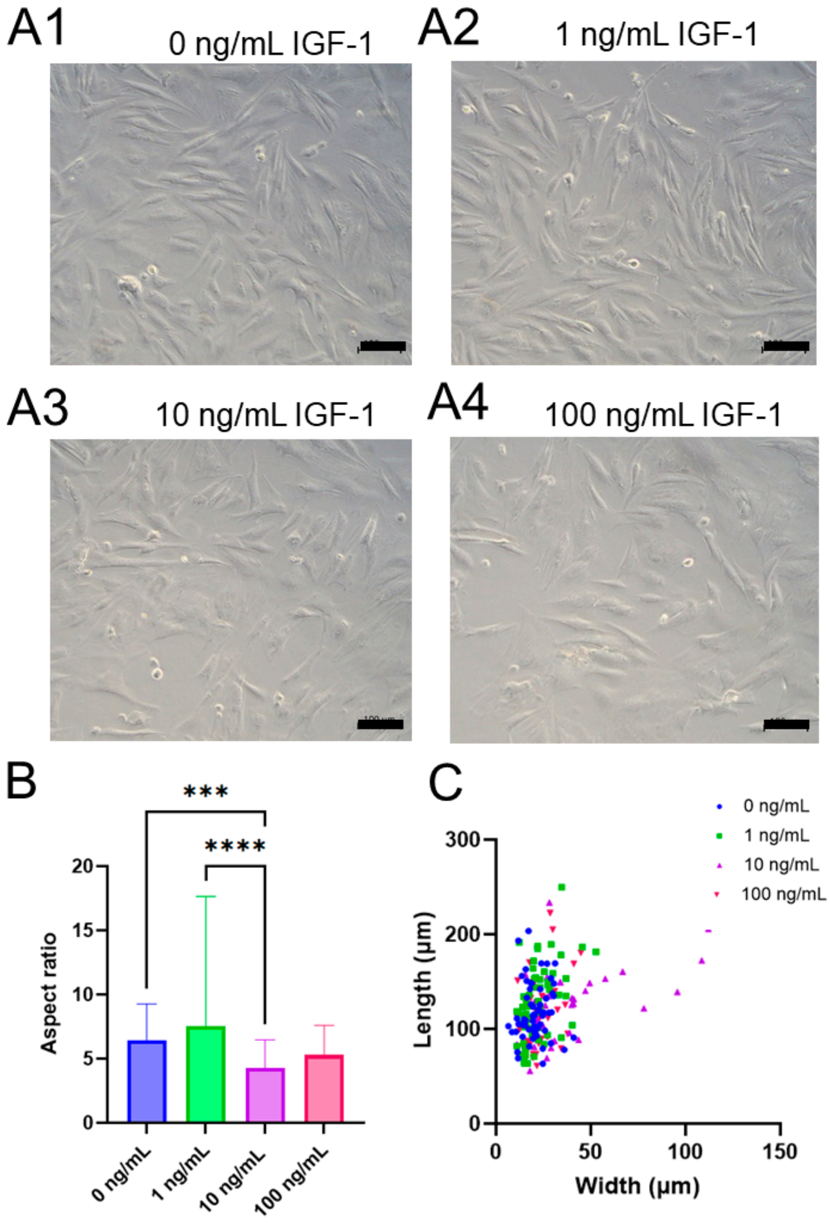

Figure S6: Aspect ratio. Rabbit Achilles tenocyte cell culture on day 3 after supplementation with IGF-1 at 1, 10 and 100 ng/mL, respectively, and control (0 ng/mL IGF-1). Microscopic images of respective conditions (**A1-A4**); aspect ratio (length: width) (**B**) and size distribution for the four conditions in a plot length versus width (**C**). p-values were considered significant if $p < 0.05$; with $p < 0.001$ (***) and $p < 0.0001$ (****).
